# Capturing judgement strategies in risk assessments with improved quality of clinical information: How nurses’ strategies differ from the ecological model

**DOI:** 10.1186/s12911-016-0243-1

**Published:** 2016-01-23

**Authors:** Huiqin Yang, Carl Thompson

**Affiliations:** 1 Centre for Reviews and Dissemination, University of York, York, UK; 2 School of Healthcare, Faculty of Medicine and Health, University of Leeds, Leeds, UK

**Keywords:** Judgement policy, Judgement strategy, Risk assessment, Clinical experience

## Abstract

**Background:**

Nurses’ risk assessments of patients at risk of deterioration are sometimes suboptimal. Advances in clinical simulation mean higher quality information can be used as an alternative to traditional paper-based approaches as a means of improving judgement. This paper tests the hypothesis that nurses’ judgement strategies and policies change as the quality of information used by nurses in simulation changes.

**Methods:**

Sixty-three student nurses and 34 experienced viewed 25 paper-case based and 25 clinically simulated scenarios, derived from real cases, and judged whether the (simulated) patient was at ‘risk’ of acute deterioration. Criteria of judgement “correctness” came from the same real cases. Information relative weights were calculated to examine judgement policies of individual nurses. Group comparisons of nurses and students under both paper and clinical simulation conditions were undertaken using non parametric statistical tests. Judgment policies were also compared to the ecological statistical model. Cumulative relative weights were calculated to assess how much information nurses used when making judgements. Receiver operating characteristic (ROC) curves were generated to examine predictive accuracy amongst the nurses.

**Results:**

There were significant variations between nurses’ judgement policies and those optimal policies determined by the ecological model. Nurses significantly underused the cues of consciousness level, respiration rate, and systolic blood pressure than the ecological model requires. However, in clinical simulations, they tended to make appropriate use of heart rate, with non-significant difference in the relative weights of heart rate between clinical simulations and the ecological model. Experienced nurses paid substantially more attention to respiration rate in the simulated setting compared to paper cases, while students maintained a similar attentive level to this cue. This led to a non-significant difference in relative weights of respiration rate between experienced nurses and students.

**Conclusions:**

Improving the quality of information by clinical simulations significantly impacted on nurses’ judgement policies of risk assessments. Nurses’ judgement strategies also varied with the increased years of experience. Such variations in processing clinical information may contribute to nurses’ suboptimal judgements in clinical practice. Constructing predictive models of common judgement situations, and increasing nurses’ awareness of information weightings in such models may help improve judgements made by nurses.

**Electronic supplementary material:**

The online version of this article (doi:10.1186/s12911-016-0243-1) contains supplementary material, which is available to authorized users.

## Background

Nurses use their judgement to identify patients at risk of deterioration in acute care. Critical care outreach systems - often nurse-led - have been implemented as a means of improving the quality of these judgements [1, 2]. Critical care outreach systems are usually initiated by track and trigger systems based on routine observations of airway adequacy, breathing and circulatory systems. Adverse observations trigger intervention and management, often in the form of expertise - such as that offered by the critical care outreach team.

In order to ensure prompt identification of patients at risk of clinical deterioration the National Institute for Health and Clinical Excellence (NICE) suggests that all hospital adult patients should receive a minimum set of physiological observations and a clear written monitoring plan at the time of admission or initial assessment [3]. These routine observations are often carried out by ward or emergency department nursing staff and they should be monitored at least every 12 h [3]. Early recognition of changes to physiological parameters is crucial to timely intervention and reducing the chances of critical events [4] and mortality. As up to 62 % of cardiac arrests are potentially avoidable [5] the evidence is that this system of identification and intervention, based largely on clinical judgement, could perform better.

Understanding the underlying mechanisms influencing information processing in nurses’ unassisted risk assessments – i.e. how nurses weigh and combine information cues to reach their judgements – would help in the design of the kinds of complex interventions (such as critical care outreach systems) likely to improve early detection and timely intervention in patients at risk of critical events or deterioration.

Critical events in patients are often preceded by changes in physiological parameters sometimes hours prior to the event [6, 7]. The National Confidential Enquiry into Patient Outcome and Death [8] suggests that approximately 66 % of patients in hospital for more than 24 h showed physiological abnormalities at least twelve hours prior to intensive care unit admission. Abnormal physiological signs are associated with patient mortality: Goldhill and McNarry [9] found a patient’s mortality risk increased with the number of physiological abnormalities (*P* < 0.001), being 0.7 % with no abnormalities, 4.4 % with one, 9.2 % with two and 21.3 % with three or more.

Evidence suggests that physiological deterioration is often unrecognised inadequately treated or dealt with inappropriately by healthcare professionals – including nurses [5, 10, 11]. Suboptimal care prior to critical care admission is relatively common, with at least 39 % of acute adult emergency patients being admitted to intensive care late in the clinical course of their illness [12]. Nurses’ failure to act appropriately is a major cause of suboptimal care: signs of deterioration in the 24 h prior to cardiac arrest in hospital were not acted on in 48 % of patients [5]. Aggressively intervening early for critical events such as myocardial infarction and shock can significantly reduce mortality [13, 14].

Understanding the underlying cognitive mechanisms of how nurses process clinical information to make risk assessments can help explain nurses’ suboptimal judgements.

This study aims to:

i) describe nurses’ judgement policies in the recognition of patients at risk of acute deterioration
ii) investigate whether their policies differ from optimal strategies derived from statistical models of the relationship between clinical information/cues and clinical outcomes (the ecology)
iii) examine whether experienced nurses’ judgement policies differ from those of novice nurses (on the basis that clinical experience has been identified as an important factor influencing clinicians’ judgement and decision making [15, 16].

With the advent of technologies such as computerised patient simulators (e.g. Laerdal SimMan™) educators can recreate far more realistic clinical judgement situations than traditional “paper-and-pen” patients - substantially enhancing the quality of clinical information for use in simulation. We also, therefore, set out to investigate the hypothesis that nurses’ judgement policies change as the quality of information in clinical simulations also changes.

## Methods

### Capturing judgement policies: Cue utilization validity & ecological validity

**Fig. 1:**
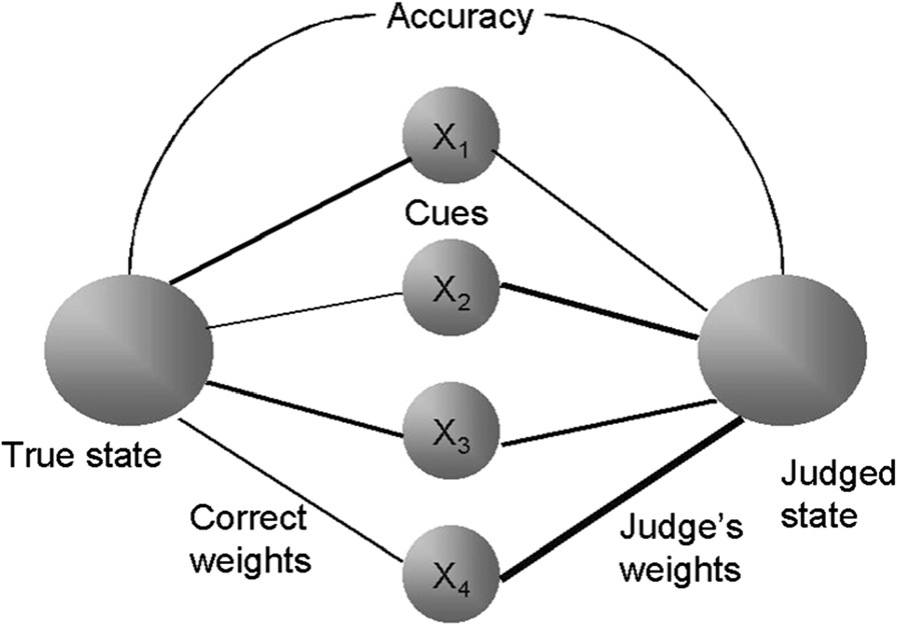
The Lens Model

Judgement analysis and the Lens Model of cognition (see Fig. 1), was used to investigate nurses’ judgement policies. This model characterises judgement as a relationship between a judgement and the information present in an environment (known as the ecology) and used in a judgement [17, 18]. It is based largely on the principles of probabilistic functionalism put forward by Egon Brunswik [19–22].

Using the Lens Model (Fig. 1) to understand judgements requires explication of three important concepts: ecological validity, cue utilisation validity, and ‘achievement’ [23]. Ecological validity refers to the correlations between proximal cues and an ecological criterion [20] (such as disease classification or level of risk). Cue utilisation validity refers to correlations between proximal cues and an individual’s judgements. Achievement refers to the correlations between the values of an ecological criterion and the values of an individual’s judgement.

In the Lens Model the left side represents the relationship (i.e. the ecological validity) between proximal cues and the ecological criterion i.e. how clinical information is correlated with an actual clinical state. The right side of the Lens Model represents the relationship (i.e. cue utilisation validity) between available cues and subjects’ judgements i.e. the importance clinicians attach to clinical information cues. Clinicians’ judgements may (and crucially, may not) be similar to the weights in the true ecology. Achievement is captured by the correlation (i.e. accuracy) between subjects’ judgements and the ecological criterion. In this paper we focus on cue utilisation validity and compare it to ecological validity: how participants use the cues in their judgements as compared to the weights in a statistical model of the judgement ecology.

### Clinical scenarios and data collection

We used five cues as recommended by NICE [3] to construct the 25 scenarios: systolic blood pressure, heart rate, respiratory rate, temperature, and levels of consciousness. The 25 clinical scenarios were randomly sampled from a large data set of patient case series (*n* = 673) [24]. Judgement ecological criteria (reference standards) were derived from the same set of patient case records: the patient being simulated was classified as at risk if the patient case was admitted to intensive care units, had cardiopulmonary resuscitation or died.

All the clinical scenarios were presented as paper cases in a booklet of clinical vignettes (see the Additional file 1 where the patient name used is a pseudonym). Natural units (such as mmHg for blood pressure and beats per minute for Heart rate) that are routinely used in the current practice were used when presenting these cues. A patient simulator (Laerdal SimMan) and bedside vital signs monitor were used to simulate the same clinical scenarios presented in the booklet of clinical vignettes. Scenarios and clinical simulations were approved by a critical care specialist nurse with more than 12 years specialist nursing experience. Nurses were asked to assess risk of a critical event independently, for each paper based scenario and then again on a data collection sheet in clinical simulations. They were asked to complete all 25 scenarios on paper cases and subsequently complete all 25 scenarios on clinical simulations. Nurses were instructed not to discuss these scenarios with each other and recorded their assessments into the data collection sheet independently. Nurses were solely asked to assess these scenarios to detect whether the simulated patient case was at risk of acute deterioration, but they were not required to take a medical intervention in response to these scenarios where the patient case was at risk of acute deterioration.

### Ethical approval

Ethical approval was obtained from the Health Sciences Research Governance Committee of the University of York, UK. All participants were given the same information about the study. All participants completed an informed consent document.

### Data analysis

#### Relative weights of validity coefficients

Relative cue weights for individual nurses in this study were derived from the cue utilisation validity (the cue-judgement correlation) and the ecological validity (the cue-criterion correlation). Relative weights can be derived from *either* regression models *or* cue validities [25]; we derived the relative cue weights for each participant from cue validities: the cue utilisation validity (cue-judgement correlation) and the ecological validity (cue-criterion correlation). Cue-judgement and cue-criterion correlations index a cue’s importance to the prediction of judgements or criteria (the ecology) [20, 26–28]. The correlations between cues and judgements indicate the emphasis judges placed on these cues; the predictive ability increases when the correlation between each cue and its dependent variables increases.

For each participant’s model the relative weights of cues were generated from the validity coefficients by normalising their absolute values to 1:

$$ r{w}_i=\frac{\left|{r}_{Ys. Xi}\right|}{{\displaystyle \sum_{i=1}^K|{r}_{Ys. Xi}|}} $$

To investigate the relative weight of the categorical variable of consciousness level, use of a single cue weight represents the judge’s overall emphasis on the information contained in the categorical cue [25]. In the analysis, the categorical cue of consciousness level was therefore put in the model together with other continuous cues to identify the single overall effect.

Cumulative relative weights were calculated from the relative weights of cue utilisation validity to assess *how much* information participants used in making their judgements. The cumulative relative weights of the third most important cue were taken as the optimal cut-off measure to evaluate the amount of information participants have used. A statistical comparison of cumulative relative weights between participants and the ecological model was also made.

Where appropriate non-parametric tests were used to detect the statistical significance of differences in relative weights between different groups since the data did not follow a normal distribution. For example, the Wilcoxon rank-sum test was used to test the significance of the difference between student and experienced nurse groups. The Wilcoxon matched-pairs signed-ranks test was used to test the significance of the difference between paper case and clinical simulations. A level of *p* < 0.05 was used as a cut-off for statistical significance.

#### Analysis of ROC curves

To identify the model’s predictive accuracy for participants’ judgements, ROC curves were generated from the aggregate models of paper cases and clinical simulations. ROC curves were obtained by plotting the sensitivity (true positive rate) against 1− specifity (false positive rate) [29]. The predictive accuracy of alternative logistic models used were assessed by comparing the areas under the corresponding ROC curves (AUC) [30]. The higher the AUC the greater the predictive performance of the model (an AUC of 0.5 would be “chance” or the equivalent of flipping an unbiased coin).

## Results

### Participants

Sixty-three students and 34 experienced nurses took part. The nurses had an average of 12 years (standard deviation (SD) 10.0) of clinical experience; the majority (*n* = 27, 81 %) were educated to diploma or first degree level. The mean age of experienced nurses was 36.6 years (SD 9.9), while the mean age of student nurses was 29.1 years (SD 9.0). 85 % of experienced nurses were female and 89 % of student nurses were female. The experienced nurses were recruited from the ward & critical care registered nurse population in hospitals in North Yorkshire. The majority of students were 2^nd^ and 3^rd^ year undergraduate nurses and registered for a diploma in nursing.

### Relative weights of Cue utilisation validity & ecological validity

#### Experienced nurses vs. Students

In paper cases experienced nurses paid more attention to consciousness level (median (Mdn) 0.259) than students (Mdn 0.240), *z* = −2.39, *P* = 0.02 and more attention to systolic blood pressure (Mdn 0.065) than students (Mdn 0.054), *z* = −1.97, *P* = 0.048. The experienced nurses paid less attention to respiration rate (Mdn 0.247) than students (Med 0.278), *z* = 2.80, *P* = 0.005. There was no significant difference in the relative weights given to heart rate and temperature by experienced nurses and students.

In clinical simulation settings experienced nurses paid more attention to consciousness level (Mdn 0.253) than students (Mdn 0.228), *z* = −3.22, *P* = 0.001 and systolic blood pressure (Mdn 0.083) than students (Mdn 0.050), *z* = −2.50, *P* = 0.01. The experienced nurses paid less attention to temperature (Mdn 0.148) than students (Mdn 0.167), *z* = 2.41, *P* = 0.02. There were no differences in relative weightings given to heart rate and respiration rate by experienced and student nurses.

#### Paper cases vs. the ecology model

In paper cases participants underused the consciousness level cue (Mdn 0.245); the ecological model suggests a figure of (Mdn) 0.301 would be more appropriate (*z* = −10.14, *P* < 0.001). They also underused respiration rate (Mdn 0.275; ecological model requires (Mdn 0.320), *z* = −7.19, *P* < 0.001), and systolic blood pressure (Mdn 0.057; ecological model (Mdn 0.079), *z* = −6.22, *P* < 0.001). In contrast participants over-relied on heart rate (Mdn 0.265; ecological model (Mdn 0.248), *z* = 3.43, *P* < 0.001), and over-relied on temperature (Mdn 0.149; ecological model (Mdn 0.052), *z* = 10.15, *P* < 0.001).

#### Physical simulation vs. the ecology model

In clinical simulation settings participants underused the consciousness level cue (Mdn 0.236; ecological model (Mdn 0.301), *z* = −8.42, *P* < 0.001); and respiration rate (Mdn 0.280; ecological model (Mdn 0.320), *z* = −4.91, *P* < 0.001), and systolic blood pressure (Mdn 0.058; ecological model (Mdn 0.079), *z* = 3.74, *P* < 0.001). Participants over-relied on temperature (Mdn 0.158) in clinical simulations (ecological model, (Mdn 0.052), *z* = 8.54, *P* < 0.001). However, there was no significant difference in the relative weights of heart rate between clinical simulation and the ecological model. Because the relative weight in the ecological model provides an optimal standard of how the participant should pay an attention to a particular cue, this non-significant difference in relative weights of heart rate between the two models indicated that the clinical simulation setting was associated with appropriate use of heart rate information.

#### Analysis of cumulative relative weights

The results showed that participants’ cumulative relative weights on the third most important cue (Mdn 0.795) in paper cases were lower than the equivalent cumulative relative weights (0.869) in the ecology model *z* = −10.14, *P* < 0.001. Similarly, participants’ cumulative relative weights on the third most important cue (Mdn 0.788) in clinical simulations were lower than the equivalent cumulative relative weights (0.869) in the ecology, *z* = −8.08, *P* < 0.001.

### Analysis of ROC curves

**Fig. 2:**
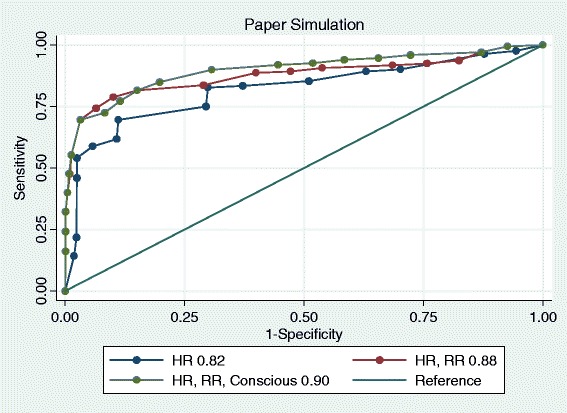
ROC curves of hierarchical models in paper cases

ROC curves were plotted for hierarchical logistic regression models for participants at the aggregate level in paper cases (Fig. 2). Each cue was sequentially entered into the models based on the ranking of the binary correlations between each cue and the dependent variable of judgements in paper cases. The AUC area with 95 % confidence interval (CI) for each model was calculated. Table 1 illustrates the model performance of the hierarchical logistic regression of judgement in paper cases.

**Table 1:** The model performance and the prediction of judgement in paper cases

Models	AUC (95 % CI)	Pseudo R2
HR	0.82 (0.80–0.84)	0.26
HR, RR	0.88 (0.86–0.89)	0.42
HR, RR, Consciousness	0.90 (0.88–0.91)	0.45
HR, RR, Consciousness, Temperature	0.90 (0.88–0.91)	0.45
HR, RR, Consciousness, Temperature, Systolic BP	0.90 (0.88–0.91)	0.45

HR Heart Rate; RR respiration rate; Systolic BP: systolic blood pressure

In the paper cases the AUC curves showed that heart rate, respiration rate and consciousness level were the three major predictors of 97 participants’ judgements. The AUC area for each model was consistent with the cumulative R-squared of each hierarchical model. Temperature and systolic blood pressure information was of no added value in predicting participants’ judgements in paper cases.

**Fig. 3:**
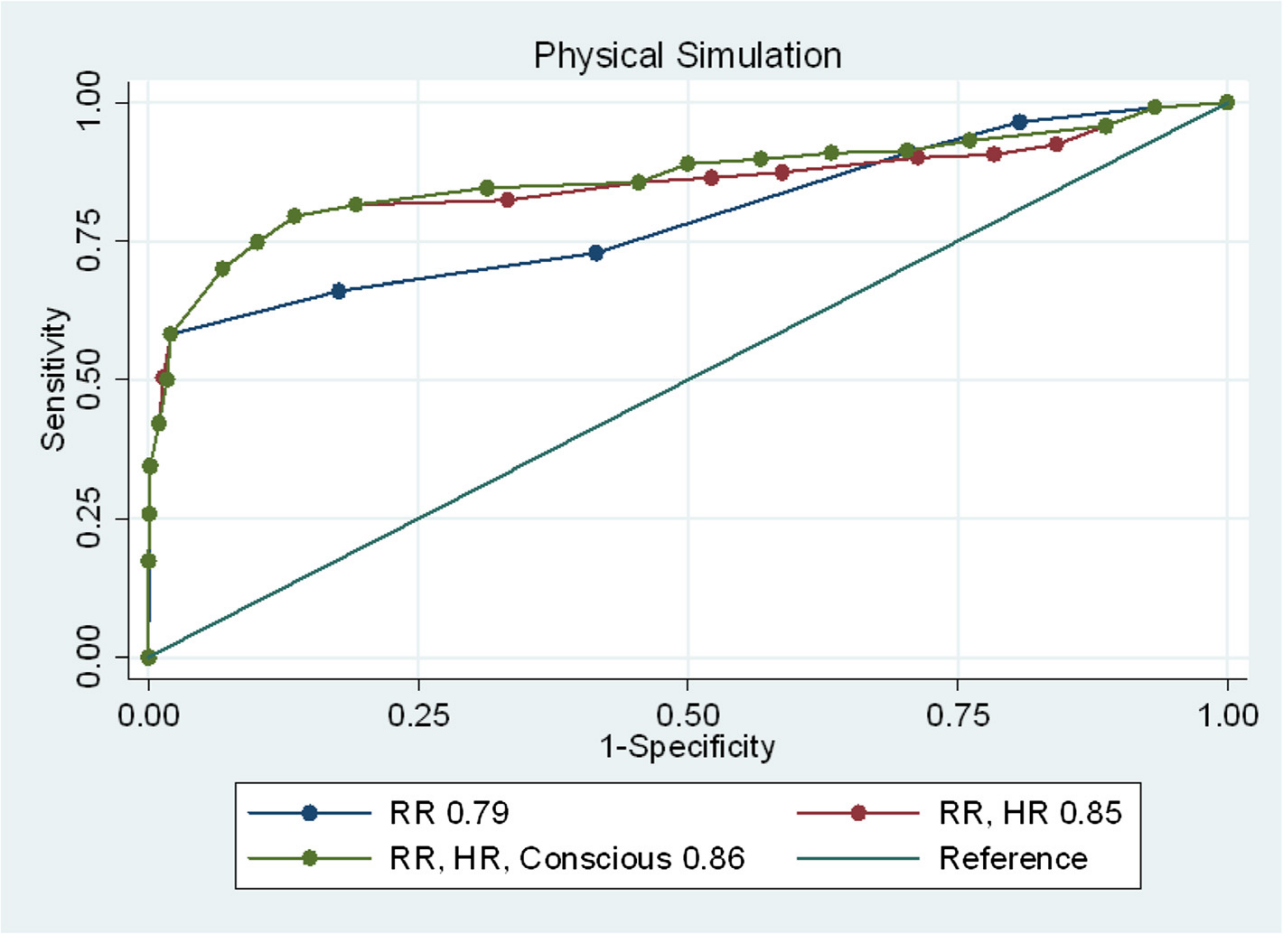
ROC curves of hierarchical models in clinical simulation

ROC Curves were also plotted for hierarchical logistic regression models for the same 97 participants at the aggregate level in clinical simulations (Fig. 3). The cues were sequentially entered into the models based on the ranking of the binary correlation between each cue and the dependent variable of judgements in clinical simulations. Table 2 shows the model performance of the hierarchical logistic regression of judgement in clinical simulations.

**Table 2:** The model performance and the prediction of judgement in clinical simulations

Models	AUC (95 % CI)	Pseudo R2
RR	0.79 (0.78–0.81)	0.28
RR, HR	0.85 (0.83–0.87)	0.37
RR, HR, Consciousness	0.86 (0.85–0.88)	0.38
RR, HR, Consciousness, Temperature	0.86 (0.84–0.88)	0.39
RR, HR, Consciousness, Temperature, Systolic BP	0.86 (0.84–0.88)	0.39

HR: Heart Rate; RR: respiration rate; Systolic BP: systolic blood pressure

In clinical simulations the ROC curves showed respiration rate, heart rate, and consciousness level were the three main predictors of participants’ judgements. It should be noted that the cumulative R-squared of hierarchical models showed that temperature may still be an additional contributor in predicting judgements of clinical simulations. However, systolic blood pressure was of no added value in predicting participants’ judgements in clinical simulation.

## Discussion

There are wide variations in individual nurses’ use of information cues (based on relative cue weights of cue utilisation validities) in paper cases and clinical simulations; a finding in line with previous research [31]. Such variations reflect the substantial differences in cue usage among individual nurses when faced with different judgement tasks.

### Judgement policies compared with the ecological model

Despite such wide variations, nurses both under-values some cues (consciousness level, respiration rate and systolic blood pressure) and put too much weight on others (heart rate and temperature) when faced with (traditional) paper based scenarios. Similar patterns were observed in the more “advanced” simulated environment of the clinical simulator (with the exception that heart rate was weighted more appropriately). Both experienced nurses and students both used more cues than needed to make appropriate judgements; this matters. Assigning similar weights to those in the ecology will improve judgement performance. For example less relevant information can reasonably be ignored [32]. If an irrelevant cue is weighted, it significantly lowers the utilisation of other relevant cues in a particular judgement, thereby resulting in an error. Providing nurses with optimal weights using models of the ecology may minimize their policy variations and improve judgement achievement.

### Judgement policies in experienced nurses and students

Cue utilisation relative weights revealed experienced nurses and students differs substantially on the cues used to make risk assessments: experienced nurses rely more on the cues of consciousness level and systolic blood pressure for risk ssessment judgements than students. These differences in relative weighting could be an important source of inconsistencies and variations in judgements between experienced nurses and nurse students.

### Judgement policies in paper cases and clinical simulation

Generally, similar patterns of relative weights of cue utilisation validity were observed in both paper cases and clinical simulations. But setting impacted on heart rate (experienced nurses over relied on it for paper cases but used it more appropriately in clinical simulations) suggesting that clinical simulation may foster a more appropriate use of at least some information. Moreover, nurses used more available information in clinical simulations than in paper cases. It is reasonable then to argue that different simulation approaches lead to variations in nurses’ judgement policies. This is in line with (or extends the logic of) studies [33–35], suggesting the format of information presentation (e.g. pictorial information is substituted for written description) significantly affects the amount of information the subjects would use. Importantly, these findings reveal that, under more natural settings where the quality of clinical information is improved, nurses use strategies that are markedly different from those elicited by paper cases.

Differences in nurse’ judgement strategies between paper cases and clinical simulations can be explained theoretically using Cognitive Continuum Theory [36]. According to this theory, pictorial cues in clinical simulations may induce more intuition in judgements, whilst more “abstract” (i.e. less visual) quantitative cues in paper cases will promote a more analytic approach. Therefore, changes in the format of information presentation may result in marked differences in judgement strategies between paper cases and clinical simulations.

The ROC curve analyses illustrate that three cues (respiration rate consciousness level and heart rate) were primarily used to reach judgements (regardless of simulation approach). This implies that nurses place more importance on these three cues in risk assessment judgements than the other two (systolic blood pressure and temperature). These findings suggest that nurses may “miss” information contained in an assessment of risk in critically ill patients.

### Strengths and limitations

In this study we used real patient cases to construct clinical scenarios which substantially enhanced the representativeness of clinical scenarios. Despite this strength, it should be recognised that cue intercorrelations are a feature of ‘vicarious functioning’ [37] in real world judgement tasks. The high levels of cue intercorrelations challenge the analysis of relative importance of cues and a large number of judgement tasks is required [38, 39]. Particularly, large numbers of judgement tasks are practically limiting: participants’ boredom or impatience as a result of judging an excessive number of tasks may influence their judgement processes [40]. In this study we derived cue relative weights using cue utilisation validity (cue-judgement correlation) and ecological validity (cue-criterion correlation), as advocated by Brunswik [20] and Hammond [26] in their early work. These relative weights of validity coefficients are able to index the independent contribution of each cue to the predictions of judgements and the ecology. This useful cue weighting approach provides a more holistic picture of how nurses value each cue compared to the ecological model.

### Implication for research

Cognitive feedback, a type of feedback describing the relations between symptoms/signs and outcomes, essentially captures the probabilistic nature of tasks and the inherent uncertainty of environment [41, 42]. This type of feedback could help improve nurses’ judgement performance. For a typical task in risk assessments, not only are nurses required to classify patients as at “risk” or “not at risk”, but also to learn the relationships between symptoms and disease outcomes. These relationships (depicted as probabilistic functionalism [26, 43]) play a significant role to help nurses gain such abilities in probabilistic inference. Providing cognitive feedbacks with task information has proved to be useful for improving judgement performance in doctors [44–47]. The potential of using cognitive feedbacks to improve nurses’ judgements should therefore be tested for future research.

## Conclusions

The findings from this study suggest that a mismatch between nurses’ judgement strategies and a pertinent ecological model may explain suboptimal judgement performance. Our findings highlight the importance of appropriate attention being paid to patients exhibiting physiological abnormalities, and correct recognition of patients at risk of acute deterioration. When making risk assessments in practice, nurses should pay more attention to important cues such as respiration rate, consciousness level and systolic blood pressure. Clinical simulations recreating real patient cases have advantages over paper based traditional approaches to simulation. These simulations and the awareness they provide can allow nurses to refine their judgement behaviours with opportunities for reflection and correction. The report by the National Patient Safety Agency [48] has revealed that insufficient training to understand the relevance of observations is one common factor contributing to patient deterioration incidents not being recognised or acted upon. Approaches such as cognitive feedback could enhance understanding of the relationship between clinical information and ecological criteria (for example, physiological signs and symptoms and true underlying risk of a critical event); it may also help nurses appreciate more about the nature of uncertainty in the probabilistic relationships that make up the provision of healthcare.

## Additional file

Additional file 1:
**Clinical background.** (DOCX 18 kb)

## Abbreviations

ROC: Receiver operating characteristic
NICE: National Institute for Health and Clinical Excellence
AUC: Areas under the corresponding ROC curves
CI: Confidence interval
HR: Heart Rate
RR: Respiration rate
BP: Blood pressure
