# Supplementary material for: Capturing judgement strategies in risk assessments with improved quality of clinical information: How nurses’ strategies differ from the ecological model
Source: BMC Med Inform Decis Mak. 2016 Jan 23;16:7. doi: 10.1186/s12911-016-0243-1 (PMC4724085; doi:10.1186/s12911-016-0243-1)
Supplement: Additional file 1: — Clinical background. (DOCX 18 kb) [file 12911_2016_243_MOESM1_ESM.docx]

**Additional file 1**

Clinical background

Mr. Robert Wright, 63 years old and 76 kg weight, was presented to the emergency room in your hospital, accompanied by his wife. He was generally feeling unwell, with a tender abdomen and vomited after each meal for past 2 days. He was born in England and he has been married for 38 years. He is a senior engineer in an automotive company. He has no food or medical allergies. There was no report of use of medications. He has no significant past medical history or history of mental illness. The details of family history are unclear. The following sets of information are available to you when you assess Mr. Wright on admission. Please make your judgements for each scenario.

An example of case scenario for risk assessment

| - Systolic blood pressure 78 mmHg - Heart rate 98 beats per minute - Respiratory rate 28 breaths per minute - Temperature 35.6 °C - Conscious level Reacting to voice | Risk  YES NO |
| --- | --- |
